# Investigating the Potential of Latent Space for the Classification of Paint Defects

**DOI:** 10.3390/jimaging11020033

**Published:** 2025-01-24

**Authors:** Doaa Almhaithawi, Alessandro Bellini, Georgios C. Chasparis, Tania Cerquitelli

**Affiliations:** 1 Department of Control and Computer Engineering, Politecnico di Torino, 10129 Torino, Italy; tania.cerquitelli@polito.it; 2 Prime Lab, Mathema s.r.l., 50142 Florence, Italy; abel@mathema.com; 3 Department of Data Science, Software Competence Center Hagenberg GmbH, Softwarepark 32a, 4232 Hagenberg, Austria; georgios.chasparis@scch.at

**Keywords:** latent space, defect detection, vehicle paint

## Abstract

Defect detection methods have greatly assisted human operators in various fields, from textiles to surfaces and mechanical components, by facilitating decision-making processes and reducing visual fatigue. This area of research is widely recognized as a cross-industry concern, particularly in the manufacturing sector. Nevertheless, each specific application brings unique challenges that require tailored solutions. This paper presents a novel framework for leveraging latent space representations in defect detection tasks, focusing on improving explainability while maintaining accuracy. This work delves into how latent spaces can be utilized by integrating unsupervised and supervised analyses. We propose a hybrid methodology that not only identifies known defects but also provides a mechanism for detecting anomalies and dynamically adapting to new defect types. This dual approach supports human operators, reducing manual workload and enhancing interpretability.

## 1. Introduction

Maintaining high-quality standards is paramount in vehicle manufacturing, spanning all stages of production, from structural integrity and engine performance to overall functionality and appearance. Among these, the paint finish holds particular importance, as it is the first attribute noticed by customers and plays a critical role in a vehicle’s market appeal (c.f. [1,2,3,4]). Achieving a flawless paint finish requires rigorous control over numerous parameters, including machinery, materials, and operator actions. As a result, quality assurance often serves as the final stage in the production cycle, tasked with identifying and addressing defects introduced throughout the process. While defect detection can follow a standardized approach, classifying defects and identifying their root causes typically depend on expert knowledge [5]. In this context, Artificial Intelligence (AI) offers significant potential to assist operators in various aspects, such as improving the accuracy of defect detection, reducing the overhead and fatigue on the operators, and expediting and streamlining production workflows.

Despite advances in AI-powered quality assurance, detecting vehicle paint defects remains a challenging problem due to several factors (c.f. [6,7]), including but not limited to the following:

- Lighting conditions: The strong lighting required for manual inspections introduces reflections that complicate automated detection systems.
- Defect characteristics: Defects are often tiny, visually similar, and diverse in type, making their automated differentiation complex.
- Temporal variability: The frequency and distribution of defects over time are generally unpredictable.
- Surface and color impact: The wide surface area of vehicles and variations in paint color affect detection processes and require nuanced feature extraction.

These challenges necessitate advanced approaches that can adapt to diverse scenarios while maintaining high detection accuracy.

Convolutional Neural Networks (CNNs) have emerged as a powerful tool for addressing such challenges (c.f. [8]) due to their ability to learn hierarchical feature representations. More specifically, using the latent spaces generated by CNNs, which provide a compressed yet highly discriminative representation of complex data, is a particularly effective method for vehicle paint defect detection [4]. They offer significant advantages in terms of re-usability and efficiency by leveraging pre-trained models, which save time and computational resources (aligned with Green AI principles) by transferring knowledge from similar cases and minimizing redundant training. Furthermore, latent spaces enhance explainability by revealing how defects are clustered and separated based on learned features, aiding operators in making informed decisions. While general latent spaces can provide valuable insights, fine-tuning them for specific use cases ensures improved performance and customization to address unique challenges. However, to overcome the limitations inherent in pre-trained models, additional supervised learning is often necessary to refine latent spaces for domain-specific applications.

This paper presents a comprehensive framework designed to support operators in detecting vehicle paint defects by leveraging and refining latent space representations generated by a pre-trained CNN-based model (selected based on quantitative comparison between MobileNet-V2, VGG16, ResNet-34 and ResNet-50). The proposed methodology begins with an unsupervised analysis of the latent space to evaluate the performance of the pre-trained model. Subsequently, a supervised fine-tuning phase is introduced to adapt the latent space to the specific characteristics of vehicle paint defects. A comparative analysis between the pre-trained and fine-tuned latent spaces highlights the impact of domain-specific customization.

We achieved high accuracy in classifying three types of defects, with the added capability to isolate samples that the model could not confidently classify for review by the operator. This feature enables an automated detection phase that reduces the manual effort by approximately ∼90%. The approach is adaptable for the few-shot learning of new defect types by repeating the supervised fine-tuning phase with a small number of samples or for enhancing the model’s ability to distinguish out-of-domain samples. This paper is structured as follows: Section 2 discusses the related work and contributions, Section 3 presents the proposed solution and methodology, Section 4 outlines the empirical settings, Section 5 provides the results and discussion, and Section 6 concludes the paper.

## 2. Related Work and Contributions

While the specific topic of vehicle paint defect detection has been addressed by only a few authors, it remains an active area of research due to the stringent quality requirements and competitive nature of the manufacturing industry. Contributions to this field can be grouped into the following three main categories: defect detection in manufacturing and quality assurance, the application of machine learning (ML) in defect detection, and the use of latent spaces for addressing detection problems.

*Defect Detection in Manufacturing and Quality Assurance*. Early work in defect detection systems focused on traditional imaging techniques and system designs. For instance, [5] describes a defect detection system implemented at Ford Spain that uses a flash-based static imaging system to analyze the shadows around defects. Reference [3] describes a surface inspection system developed at Volkswagen’s Wolfsburg plant that improves paint defect detection using image processing techniques. Reference [1] proposes a system for detecting defects on semi-specular and painted surfaces using a robotic arm equipped with camera equipment, tested in controlled laboratory settings with vehicle parts provided by an automotive OEM.

Despite advances in image processing techniques, these systems often struggle with challenges such as complex backgrounds, noise, and varying lighting conditions, as highlighted by [9]. These limitations have driven the adoption of deep learning-based methods, which take advantage of advances in computing power and digitization to overcome traditional shortcomings.

*Machine Learning in Defect Detection*. The application of ML to defect detection in automotive manufacturing has gained traction in recent years. Reference [10] explores defect prediction by combining contextual information with time series analysis, highlighting the adaptability of ML techniques to various manufacturing processes. Reference [4] developed a system that uses ant colony optimization to detect edges, eliminate reflections, and identify five types of defects, including scratches, drops, and raindrops. Reference [8] extends the previous work by creating a dataset of 2468 images containing the following seven types of defects: bubbles, dust, fouling, pinholes, sagging, scratches, and shrinkage. This method then applied convolutional models such as MobileNet-V2, VGG16, and ResNet-34 to carry out the detection tasks. Reference [7] proposes a mobile-transformer algorithm for car body defect detection and compares its accuracy with Vision Transformer (ViT), MobileNet-V2, VGG16. These works demonstrate the value of ML models in addressing the challenges of defect variability and lighting conditions. Finally, reference [2] recognizes defects using a one-against-all SVM classifier.

*Latent Spaces in Detection Problems*. Latent spaces generated by pre-trained CNNs have recently emerged as a key focus in defect detection, addressing challenges like class imbalance and feature extraction. For instance, [11] uses latent space representations to generate new samples for unbalanced ship coating defect classification, effectively mitigating the challenges posed by the limited data. Reference [12] modifies the latent space of a pre-trained CNN to facilitate automatic image generation and labeling, demonstrating its utility in object detection tasks.

*Our Approach.* Despite the advancements in defect detection systems, most existing studies lack publicly available datasets or detailed descriptions of data collection methods. This limitation complicates reproducibility and benchmarking in industrial applications. Another aspect is that the existing research focuses on the pure benchmarking of different solutions used for classification and/or detection, which produces a static system that needs an amount of new labeled data to be reconstructed each time a new defect occurs with no handling of mislabeled defects.

While some studies have explored defect detection using CNNs (c.f. [7,8]), the explicit investigation of latent spaces remains unexplored, particularly for painted surfaces. Previous studies have focused on either benchmarking architectures or developing supervised classifiers for specific defect types. Few works, if any, have investigated the potential of latent space representations to enhance both accuracy and explainability in defect detection tasks in general (c.f. [2,10,11,12]). Furthermore, the application of latent space analysis to paint defect detection—a domain where explainability is essential for operator trust and process improvement—has not been comprehensively addressed.

Therefore, this paper aims to fill this gap by introducing a novel framework that leverages latent space representations to improve explainability in defect detection. The main contribution of this work is the investigation of the potential of latent space in providing flexibility, scalability, and interpretability in defect detection tasks while maintaining the accuracy of commonly used architectures (such as CNNs). A preliminary idea of this approach was introduced by [6]. In this paper, instead, we expand on the methodology, experiments, results, and discussions to provide a comprehensive study while a novel framework is introduced.

Therefore, our contributions can be summarized as follows:

1. Investigating latent spaces potential: We investigate how latent space representations can be analyzed and utilized to provide insights into defect clustering, anomaly detection, and the separability of defect types.
2. Combining supervised and unsupervised approaches: By integrating supervised fine-tuning with unsupervised latent space analysis, we propose a hybrid methodology that adapts dynamically to new defect types while maintaining high accuracy for known defects.
3. Focusing on CNN-based architectures: We demonstrate that CNNs, with their ability to capture hierarchical feature representations, are particularly well suited for latent space exploration and defect detection.
4. Application to paint defect detection: To our knowledge, this is the first work to explicitly analyze and utilize latent spaces for explainability and adaptability in the context of painted surface defect detection.

## 3. Materials and Methods

As mentioned before, detecting defects is very challenging and stressful for operators, especially for their vision, as they need to look for tiny defects on a very big surface in an extremely bright environment for long hours. In this work, we aim to support the operators, but not replace them, using AI to reducing the surface/defects they need to check. To do so, we chose a very powerful pre-trained model (namely ResNet50) as our backbone to extract features from defect images and then analyze, visualize, and cluster these features using the latent spaces methods and techniques. In this section, we propose an overview of our proposed solution in Section 3.1, the motivation in Section 3.2, the details of our methodology in Section 3.3, and the unsupervised techniques used and the evaluation metrics in Section 3.4 and Section 3.5, respectively.

### 3.1. The Proposed Solution

**Figure 1:**
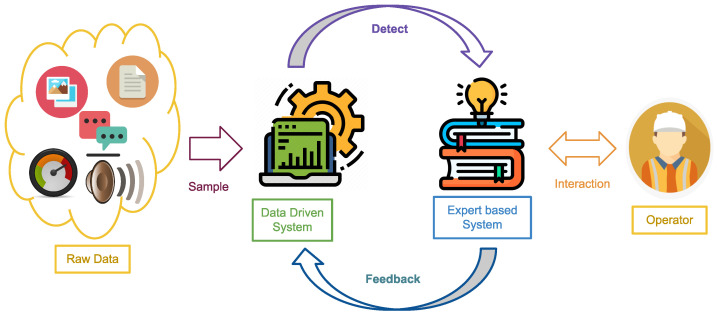
Proposed solution architecture.

Data-driven methodologies are widely used to extract useful and hidden knowledge to support decision-making processes [13]. The proposed solution (shown in Figure 1) consists of the following two main components:

- Data-driven system: AI model that takes images as input and outputs the label of the detected defect
- Expert-based system: Interacts with the human operator by displaying the detection of the previously mentioned component and providing the correct label taken from the operator

In this paper, we focus on the data-driven system, and we simulate the expert-based part by the final analysis of the outliers.

### 3.2. Motivation

The study of latent spaces in machine learning has gained significant attention for its potential to uncover meaningful patterns and structures within data. These high-dimensional representations encode the relationships between data points, offering insights that are crucial for tasks such as clustering, classification, and anomaly detection. In this work, we aim to explore the potential of latent spaces in enhancing defect detection systems, particularly in scenarios where subtle and complex defects arise. By investigating these spaces, we seek to understand how they can be leveraged to improve system performance and adaptability.

To achieve this, we focus on CNN-based models, which are well suited for capturing hierarchical and discriminative features in image data. CNNs provide powerful latent representations that enable the identification of intricate patterns, making them ideal for applications where precision and interpretability are critical. Their ability to learn meaningful features from raw data positions them as a key tool for our investigation into latent spaces.

The overarching purpose of this article is to support human operators in defect detection processes, not to replace them. Human expertise is invaluable in industrial settings, but operators often face an overwhelming volume of data they need to analyze. To alleviate this burden, our work aims to reduce overhead in the following two key ways:

- Minimizing human involvement: By filtering out the majority of samples that do not require human attention, the system can focus operator effort on the most ambiguous or critical cases.
- Adapting dynamically to new defects: Our approach incorporates a dynamic system that can quickly adapt to new types of defects or anomalies with minimal effort, ensuring it remains effective even in evolving environments.

One of the key challenges we address is the limitation of relying solely on either supervised or unsupervised techniques. Supervised methods excel in accurately identifying known defect types, but they struggle with dynamic environments where new defect types emerge. On the other hand, unsupervised techniques are effective in detecting anomalies and uncovering new patterns but lack the precision required for known defect classification. Therefore, we propose a hybrid approach that combines the strengths of both techniques as follows: leveraging supervised learning for accurate classification of known defects and unsupervised methods for dynamic adaptability to new defect types or anomalies. This dual strategy ensures a robust, scalable, and operator-friendly solution that bridges the gap between accuracy and adaptability in defect detection.

**Figure 2:**
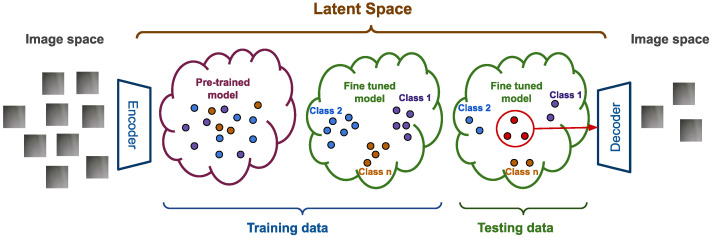
The concepts of our methodology.

Figure 2 illustrates the key concepts of our proposed methodology, progressing from the image space to the latent space using a pre-trained model as an encoder. Initially, an unsupervised analysis reveals that the latent representations in the unrefined latent space are not well separated. This is expected, as the pre-trained model was trained on a generic and diverse dataset, which may not align closely with the specific characteristics of the studied data.

To address this, a supervised training phase is conducted to fine-tune the model using a clean, labeled training dataset. This refinement process adapts the model and its latent space to the specific task, resulting in the better separability of the labeled classes and a more tailored latent space representation.

Once fine-tuned, the model is used to encode unseen data (the testing dataset) into the refined latent space. An unsupervised analysis of this new latent space reveals the following two distinct sets of samples: one set where the samples exhibit a high confidence of belonging to specific classes, due to their strong alignment with the class features in the latent space, and another set where the samples are less clear, exhibiting ambiguity or inconsistency. The final goal is to trigger an automated labeling approach for the high-confidence set while redirecting the ambiguous samples back to the image space for manual verification, ensuring both efficiency and accuracy in the defect detection process.

### 3.3. Methodology

The most common systems used for defect detection rely on image processing, where a model is trained on a sufficiently labeled dataset to detect defects in new data. However, to train an effective AI system with a relatively small amount of data, our approach seeks to minimize the required dataset size by shifting from raw image pixels to the latent space of a pre-trained model. These latent spaces are constructed and refined during the training phase, and the modified space is then used for the inference of new samples.

**Figure 3:**
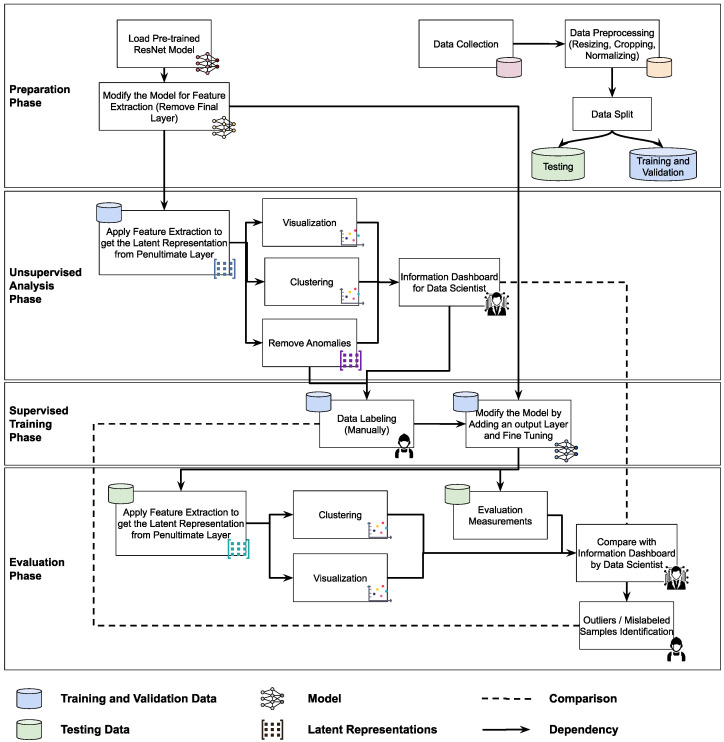
Our methodology consists of four main phases, where the icon at the top left of each block represents the input and the icon at the bottom right indicates the output type.

Figure 3 illustrates the detailed steps of our methodology, which can be categorized into the following four main phases:

- Preparation phase: This phase involves collecting defect image data, followed by pre-processing steps such as resizing, cropping, and normalizing the images. The dataset is then split into training/validation and testing sets (more details on the dataset are provided in Section 4.1). For the model, we begin by loading the pre-trained ResNet model and removing the final layer, preparing it to function as a feature extractor (more details on the model are provided in Section 4.2).
- Unsupervised analysis phase: In this phase, we extract the latent representation from the penultimate layer of the model and perform an unsupervised analysis using techniques such as visualization, clustering, and anomaly detection (further details on the methods used are provided in Section 3.4). The outcome is an information dashboard for data scientists, offering deeper insights into the data distribution and relationships within the latent space.
- Supervised training phase: This phase requires a labeled dataset for supervised training. It begins with data labeling, followed by modifying the model architecture to fine-tune it. A linear layer with one neuron per class is added, and the model is trained on the training dataset (details on the fine-tuning process can be found in Section 4.3).
- Evaluation phase: In this phase, we validate the model performance using traditional evaluation metrics (details on the metrics used are provided in Section 3.5). Additionally, we conduct some of the unsupervised analysis (visualization and clustering), again using the new latent space to compare the results, ultimately identifying any outliers or mislabeled samples.

To apply the methodology described earlier, we used a standard dataset of vehicle painting defects available online, along with the ResNet-50 model as the base encoder for the latent space (more details can be found in Section 4). The following section outlines the unsupervised techniques used and the evaluation metrics applied.

### 3.4. Unsupervised Techniques

Although unsupervised techniques were traditionally used when labels were unavailable or difficult to assign, they are now commonly employed to gain a deeper understanding of the data and uncover hidden relationships. As shown in Figure 3, we apply several of these techniques for different purposes, including visualization, clustering, and anomaly detection. In this subsection, we provide a more detailed description of these techniques.

#### 3.4.1. Visualization

Visualization of high dimensional data is primarily achieved using Dimensionality Reduction Algorithms (DRAs). These techniques serve various purposes, such as feature extraction [14], data visualization [15], pattern recognition [16], or even acting as a pre-processing step [17].

One of the most well-known and widely used DRAs is isometric mapping (ISOMAP), discussed in [18]. This classic nonlinear projection-based algorithm focuses on preserving the global structure of the data. More recent algorithms, such as t-distributed stochastic neighbor embedding (t-SNE) [19] and Uniform Manifold Approximation and Projection (UMAP) (cf. [20,21]) are designed to retain more information in reduced dimensions when the local geometry approximates Euclidean space. On the other hand, PaCMAP Pairwise Controlled Manifold Approximation Projection, introduced in [22], is a newer algorithm that optimizes both global and local structures.

To validate our work, we compare the results using ISOMAP, UMAP, and PaCMAP to analyze the nature of the relationship within the latent space and to check whether there is a more global or local structure in the distribution of the samples (the results can be found in Section 5).

#### 3.4.2. Clustering

We apply clustering algorithms like K-means or DBSCAN on the latent vectors to detect natural groupings within the latent space which can reveal clusters corresponding to distinct defect types or similarities between types. In the latent space, images of defects are represented by feature vectors, where similar types of defects should naturally cluster together based on their shared characteristics. By clustering these latent representations using algorithms like K-means and DBSCAN, we can identify groups of similar defects even if they are unlabeled.

DBSCAN (Density-Based Spatial Clustering of Applications with Noise), first presented in [23] and then revisited in [24], is a clustering algorithm that groups data based on density, making it particularly useful when the latent space has clusters of various sizes or shapes. Unlike K-means, DBSCAN does not require specifying the number of clusters upfront, and it can effectively label low-density points as noise, which can help in identifying new, distinct defect types as outliers.

#### 3.4.3. Anomaly Detection

This analytical step performs anomaly detection to identify patterns in the data that deviate from the expected behavior in the latent space. Specifically, anomalies in the latent space may indicate new types of defects, which can be flagged for further review and potential classification. To identify potential outliers in the latent space, in addition to DBSCAN, we use Isolation Forests [25].

Isolation Forests is an anomaly detection algorithm that uses binary trees to isolate anomalies. The core idea of the algorithm is to randomly select a feature and then choose a value to split the data between its maximum and minimum values. This process effectively isolates anomalous data points more quickly than other methods, making it particularly efficient for large datasets.

### 3.5. Evaluation Metrics

In addition to using visualization techniques to compare results, we apply both unsupervised and supervised evaluation metrics.

For the unsupervised analysis, we use the Adjusted Rand Index (ARI) to assess clustering performance. This metric compares the clusters to known defect labels to determine whether the clusters correspond to actual defect types [26]. Another metric is the Silhouette Score, which measures clustering quality by evaluating how similar a point is to its own cluster compared to other clusters [27]. We use this score to identify the best distance metric that the latent space might have encoded.

For the supervised phase, we rely on accuracy to interpret the results of the validation and analyze the model’s performance. Accuracy is calculated as the ratio between the number of correctly predicted labels and the total number of samples in the validation set. Additionally, we use the confusion matrix when needed to identify which classes the model may be confusing.

## 4. Empirical Settings

One of the main challenges in this case was finding a suitable dataset for our analysis. However, we found a small image dataset that supports multi-defect detection. With some pre-processing and modifications, we adapted it for our study. In this section, we detail the experimental setup, starting with the dataset and pre-processing steps in Section 4.1, followed by the pre-trained model used in Section 4.2, the supervised training process in Section 4.3, and implementation details in Section 4.4.

### 4.1. Dataset and Pre-Processing

**Figure 4:**
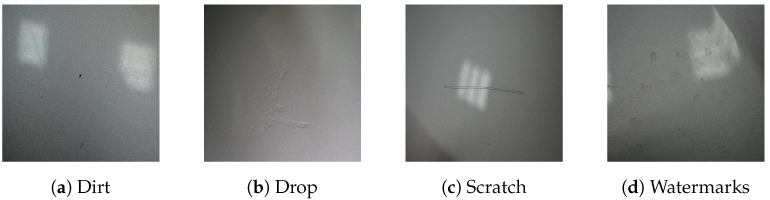
Data samples representing dirt, drop, scratch, and watermarks.

The dataset consists of images of vehicle paint defects, with a total of 344 images, each sized at 640×640. These images are divided into 240 for training, 70 for validation, and 34 for testing. The dataset includes four types of defects as follows: dirt, drops (named initially “runs”), scratches, and watermarks. Figure 4 shows an example of each class. We pre-processed the data as follows: :

- We resized the images to 224×224 to be compatible with the model input.
- For the training samples, we applied a random horizontal flip to slightly increase the challenge for the model during training.
- We normalized the pixel values, which is a standard step in image processing to mitigate the impact of high or low pixel values.

We began the unsupervised analysis using the entire training dataset. For the supervised phase, however, we manually curated and labeled the dataset to focus on specific defect types. The original dataset was designed for multi-defect detection, so we narrowed our focus to the following three defect classes: dirt, drop, and scratch. This decision was made because the available samples for watermarks were too few and often contained overlapping defects within the same image. As a first attempt, we excluded images with multiple evident defects.

After this refinement, the training dataset was reduced from 240 images to 201. The final labels were assigned as follows: dirt = 0 (36 samples), drop = 1 (83 samples), and scratch = 2 (82 samples). The original dataset is available at [28]. For model validation and testing, we retained the original validation and test datasets, which contained 70 and 34 images, respectively. This approach ensured that the model was tested for robustness against outlier samples.

### 4.2. ResNet-50

We selected ResNet-50 [29] for its ability to create deeper and richer latent spaces, enabling superior defect detection and classification in vehicle paint applications. Compared to ResNet-34 [8], ResNet-50, with approximately ∼25.6 million parameters, offers better hierarchical feature extraction, higher accuracy, and improved latent space separability. Despite its slightly higher computational cost, ResNet-50’s advantages make it the preferred choice for robust and scalable solutions in detecting subtle and diverse defects in industrial quality assurance processes.

The ResNet-50 model has a 50-layer deep Residual Neural Network (ResNet) architecture originally developed in 2015 for image recognition applications [29,30]. It remains widely used in the field of image processing. The ResNet architecture is built on repeated residual blocks, where each block functions as a small neural network. A unique feature of ResNet is the use of residual (or skip) connections, where the input of a block is added to its output before being passed to the next block. This approach mitigates the vanishing gradient problem and facilitates the training of very deep networks. Similar residual connections are employed in other architectures, such as the original LSTM network [31] and transformer models [32].

**Figure 5:**
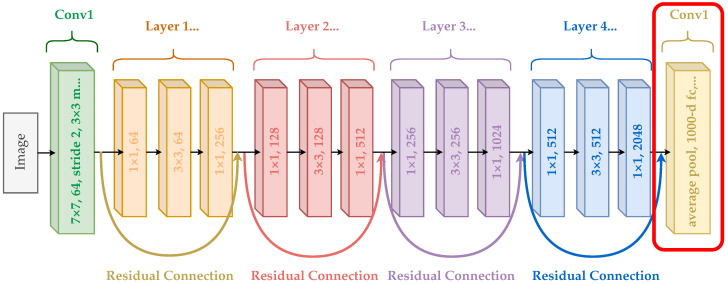
Original ResNet-50 architecture. The last layer (in the red box) was removed in the feature extraction settings and the latent representations were extracted using the penultimate layer.

The architecture of ResNet-50 is illustrated in Figure 5. The model comprises 48 convolutional layers organized into 16 residual blocks, each consisting of three layers and a residual connection. These convolutional layers are flanked by a max pooling layer at the input and an average pooling layer at the output.

Pre-trained ResNet models are widely utilized, often with customized modifications to suit the specific requirements of a given case study. For instance, ResNet-50 has a pre-trained version available in the torchvision library [33], trained on the ImageNet dataset. The ImageNet dataset includes 1,281,167 training images, 50,000 validation images, and 100,000 test images spanning 1000 distinct classes. In its original configuration, the ResNet-50 model has an output layer with 1000 nodes, each corresponding to a class. This pre-trained model is frequently employed as a backbone or feature extractor.

Since ImageNet is a highly generic dataset, the model’s output layer typically needs to be adapted to match the number of classes relevant to the case study. This adaptation can be achieved using the following two approaches to transfer learning:

- Fine-tuning: This involves updating all model parameters using the training dataset, effectively retraining the entire model.
- Feature extraction: In this approach, only the final layer’s weights are updated to predict the labels, while the pre-trained model is a fixed feature extractor.

In this work, we utilize ResNet-50 in both modes. Initially, the model is employed as a feature extractor by removing its final layer to generate latent representations of the data points using the penultimate layer output which consists of 2048 nodes. These representations are then used for unsupervised analysis and to evaluate the supervised training phases. Additionally, we fine-tune the model during supervised training. For this purpose, we modify the architecture by replacing the output layer with a new one containing three nodes, corresponding to the three classes in our dataset, dirt, drop, and scratch.

### 4.3. Supervised Training

During the fine-tuning phase, the original output layer of the ResNet-50 model, which consisted of 1000 nodes (configured for ImageNet classification), was replaced with a linear layer of three nodes. This new layer was tailored to the number of classes in the training dataset (dirt, drop, and scratch), ensuring compatibility with the task-specific classification requirements. The modified architecture is illustrated in Figure 5, where the replaced layer is highlighted by a red box.

The fine-tuned model was trained over 100 epochs with a batch size of 8 images on the training dataset (201 images in total). Training optimization was performed using the Stochastic Gradient Descent (SGD) optimizer, configured with a learning rate of 0.001 and a momentum of 0.9. The optimizer is applied using the cross-entropy loss function to evaluate and adjust the model’s parameters during training (the implementation was via PyTorch, see [34] for details).

### 4.4. Implementation Details

Experimental validation was carried out with Google Colab with T4 GPU settings and a Python kernel. The PyTorch library [34] was utilized for the essential training components, including the loss function, optimizer, and output layer modification. The torchvision library [33] was used to download the pre-trained ResNet-50 model and to apply image transformation and pre-processing operations.

For clustering, anomaly detection techniques, and evaluation measures, the Scikit-learn library [35] was employed. Visualization tasks were performed using dimensionality reduction techniques, including ISOMAP and UMAP from Scikit-learn [35] and UMAP-learn [36], respectively, as well as PaCMAP from the PACMAP library [22].

## 5. Results and Discussion

In this section, we demonstrate the obtained results of the Sensitivity, Unsupervised, and Supervised Analysis in Section 5.1, Section 5.2, and Section 5.3, respectively, and then, we discuss these results in Section 5.4.

### 5.1. Sensitivity Analysis

A sensitivity analysis was conducted to choose the most convenient unsupervised and supervised techniques to be used.

Table 1 shows a comprehensive analysis of the Silhouette Score for the latent space of ResNet-50, based on K-means clustering, to compare different distance metrics on the training dataset before fine-tuning.

The highest scores were observed when using cosine and correlation distances (0.18 each) and squared Euclidean distance (0.2). These results suggest that the latent space may not adhere to a strictly Euclidean geometry. Some limitations of K-means include the fact that it is restricted to the use of Euclidean distance and the number of clusters must be specified in advance. To further investigate how the choice of spatial metric affects clustering quality, we used DBSCAN, which does not require a prior number of clusters, can use a metric parameter as distance, and detects anomalies in the distribution of data points.

Although the use of DBSCAN was not helpful in labeling the points, we combined the anomalies detected by DBSCAN using cosine distance with Isolation Forests to remove these anomalies from the training dataset. This resulted in the exclusion of a total of twelve samples, as only two samples overlapped with the two methods.

In addition, a comparative analysis was conducted to compare ResNet-50 with other CNN-based architectures, namely, MobileNet-V2, VGG16, and ResNet-34 (presented in [8]). The same steps described in Section 4.3 were applied on the other three pre-trained models. As shown in Table 2, MobileNet-V2 exhibited faster performance, while ResNet-50 achieved the highest classification accuracy on the validation set. These results validate our choice of ResNet-50 for post-production quality assurance, where accuracy is essential and latent space separability is better represented.

### 5.2. Unsupervised Analysis

The unsupervised analysis is a very important step as it provides more information on the data distribution and relationships at a lower cost (in terms of resources and time) than supervised fine-tuning. In this analysis, we exclusively focused on the training data. After extracting features using ResNet-50, we obtained a dataset of 240×2048 latent vectors (240 images, each represented by 2048 features as the output of the penultimate layer). These latent vectors were visualized in a 2-dimensional space using the following three different Dimensionality Reduction Algorithms (DRAs): ISOMAP, UMAP, and PaCMAP. The resulting visualizations are presented in the first row of Table 3. The second row shows the same visualizations, but the points are colored based on the K-means clustering results with k=4.

While applying K-means clustering directly to the full latent representations (240×2048) did not reveal a clear separation of the classes, the PaCMAP visualization in two dimensions highlighted three distinct clusters. To investigate further, we applied K-means clustering with k=3 to the dimensionally reduced representations obtained from PaCMAP. This yielded three clusters. Upon manually examining the corresponding images within each cluster, we found that all defect types occurred in all three clusters, rendering this approach unhelpful in the labeling phase. However, there may still be valuable information, particularly from the UMAP and PACMAP results, that needs to be further analyses to confirm whether these separate regions have different probability distributions, each belonging to a cluster, and whether this separation may be meaningful.

### 5.3. Supervised Analysis

The fine-tuned model was used as a feature extractor to generate new latent features for the validation dataset, resulting in 70×2048 latent vectors (70 images, each represented by 2048 features as the output of the penultimate layer). Table 4 presents the visualization of these latent vectors in 2D using Dimensionality Reduction Algorithms (DRAs). The first row shows the latent vectors with their actual labels, while the second row shows the same vectors colored by K-means clustering results.

The visualization reveals that three clusters (0, 2, and 3) are separated, while cluster 1 is centrally located, as highlighted by ISOMAP. Cluster 1 likely contains anomalies or outliers. To verify this, we manually reviewed the images within each cluster. It was confirmed that: (a) Cluster 0 corresponds to scratch samples; (b) Cluster 2 corresponds to dirt samples; (c) Cluster 3 corresponds to drop samples; and (d) Cluster 1 contains ambiguous samples, such as those with watermarks or multiple defects (these samples are detailed in Appendix A).

The Adjusted Rand Index (ARI) for the K-means labels was calculated to be 0.89, reflecting a strong alignment exists between the clustering results and the actual labels.

This analysis on the validation set has demonstrated a mechanism for automated labeling, where the anomaly cluster (cluster 1) identifies samples requiring manual review by an operator. Notably, this cluster comprises only 11% of the entire dataset, significantly a reduction in the manual effort needed. Applying this K-means model to the testing set (34 images) resulted in the identification of three samples as anomalies, which also require manual inspection.

### 5.4. Discussion

The results of the unsupervised and supervised analyses reveal important insights into the role of latent space representations in defect detection and the challenges associated with clustering and anomaly detection. In the unsupervised analysis, the initial latent space extracted from ResNet-50 (240 × 2048 latent vectors) provided attached clusters with no definitive classes in each when directly clustered using K-means, as indicated by the low Silhouette Scores (∼0.2 for squared Euclidean distance and ∼0.18 for cosine distance). This suggests that the latent space is not entirely Euclidean and requires careful metric selection. Dimensionality reduction algorithms (PaCMAP, UMAP, and ISOMAP) on the other hand, highlighted the structure, with PaCMAP showing three distinguishable clusters. However, further investigation revealed that these clusters did not correspond to distinct defect types, highlighting the difficulty of achieving meaningful class separation without fine-tuning.

Clustering approaches like DBSCAN and Isolation Forests were also applied to detect anomalies. DBSCAN with cosine distance identified six anomalies, while combining it with Isolation Forests reduced false positives to twelve removed samples. The removal of these anomalies improved the overall data quality, laying the foundations for the supervised phase.

Although the unsupervised analysis alone was not sufficient, it helped us to understand the degree of interdependence within the different classes and to eliminate some anomalies that may confuse the subsequent fine-tuning process, as repeating the fine-tuning process on such pre-trained models is very expensive, both in terms of resources and time.

In the supervised phase, fine-tuning the model on a curated dataset significantly enhanced performance, achieving a validation accuracy of 92.31%. Visualizing the new latent features of the validation dataset in two dimensions revealed well-separated clusters that correspond to the defect types (dirt, drop, and scratch). Notably, Cluster 1, which appeared mixed in the unsupervised analysis, predominantly contained samples with multiple defects or anomalies like watermarks, aligning with the manually confirmed findings. This validation step achieved a high Adjusted Rand Index (ARI) of 0.89, underscoring the reliability of the fine-tuned model in separating defect types.

Additionally, the clustering results demonstrated the practicality of the proposed approach for automatic labeling. The mixed cluster (11% of the data) was identified as requiring a manual review, streamlining the operator’s task while maintaining high accuracy. Applying the same model to the testing dataset yielded three potential anomalies requiring manual verification, demonstrating the method’s scalability and robustness.

In summary, the combination of fine-tuning and latent space analysis proved effective in improving clustering quality, anomaly detection, and overall defect classification accuracy. These results highlight the importance of customizing the latent space to domain-specific challenges and suggest a practical workflow for integrating automated and human-led quality control in industrial applications.

## 6. Conclusions

In this study, we present an approach for detecting paint defects on vehicles using image datasets and a ResNet-based model. Our methodology involved analyzing the latent representations generated by a pre-trained model to classify the following three distinct defect types: dirt, drop, and scratch, achieving promising accuracy. By examining the latent space, we gained deeper insights into the relationships and distribution of the dataset samples, which facilitated the identification of mislabeled data and paved the way for the seamless integration of additional defect categories. Analyzing the latent space of this case allowed us the flexibility, scalability, and precision required to ensure a robust solution for AI-driven quality control in manufacturing environments is established.

In addition, the solution can adapt to new defect types as the latent space methodology allows the system to recognize and classify emerging or previously unseen paint defects with minimal manual intervention. This adaptability is particularly useful in production environments where new types of defects may arise due to changes in materials, environmental conditions, or manufacturing processes.

Therefore, for future work, we aim to conduct further comparative analysis to incorporate newer method,s such as vision transformers or a faster R-CNN as backbone models, and more sophisticated clustering techniques. Future research will also focus on incorporating few-shot learning techniques for the addition of new defect types and exploring the handling of out-of-domain samples automatically. In addition, we plan to develop this methodology into a comprehensive framework suitable for integration into manufacturing systems using more data samples, either by collecting real datasets or generating synthetic samples .

## Figures and Tables

**Table 1:** Silhouette Score of the latent representations and labels of the training dataset before fine-tuning. The underlined numbers represent higher values.

euclidean/l2	cosine	cityblock/l1/manhattan	braycurtis	canberra	chebyshev
0.12	0.18	0.11	0.11	0.066	0.095
correlation	dice	hamming	jaccard	minkowski	rogerstanimoto/sokalmichener
0.18	−0.057	0.0064	−0.056	0.12	−0.052
russellrao	seuclidean	sokalsneath	sqeuclidean	yule	nan euclidean
−0.084	0.074	−0.052	0.205	0.053	0.12

**Table 2:** Comparative analysis with other CNN-based architectures to compare accuracy after 100 epoch and the time of each epoch in seconds. The underlined numbers represent best values.

	ResNet-50	MobileNet-V2	VGG16	ResNet-34
Accuracy	100.0%	97.8%	96.7%	97.8%
Epoch time (s)	6.04	3.65	7.88	4.84

**Table 3:** Visualization of the latent representations of the training data in 2D using Dimensionality Reduction Algorithms (DRAs): ISOMAP, UMAP, and PaCMAP. The first row displays the general data distribution, while the second row shows the same data, colored according to K-means clustering results with *k* = 4.

ISOMAP	UMAP	PaCMAP
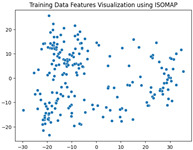	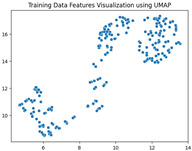	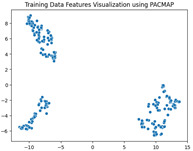
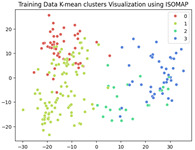	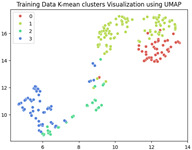	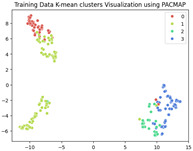

**Table 4:** Visualization of fine-tuned latent representation of the validation data in 2D using the DRAs (ISOMAP, UMAP, and PaCMAP), with the first row representing the data in general and the second row colored by the K-means of 4 clusters.

ISOMAP	UMAP	PaCMAP
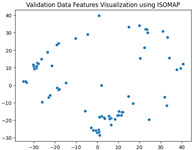	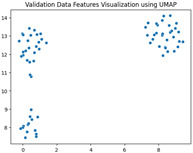	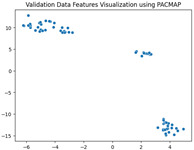
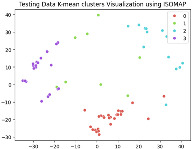	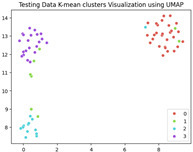	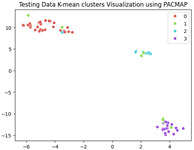

**Figure A1:**
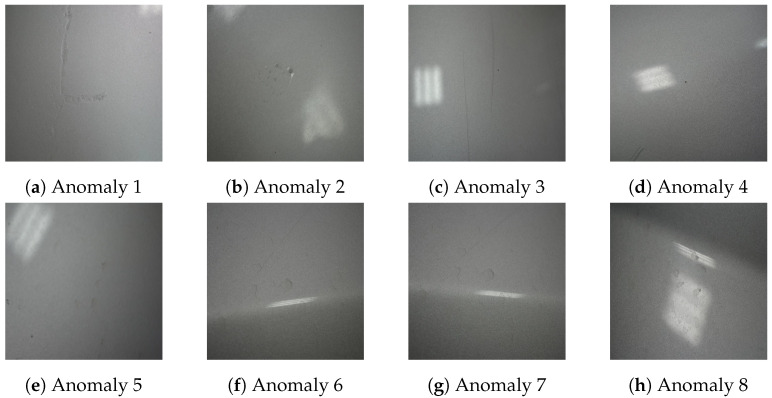
Validation data anomaly samples.

## Data Availability

The original data is available at https://universe.roboflow.com/poli-h7nww/final-year-car-paint-defect (accessed on 23 January 2025).

## Abbreviations

The following abbreviations are used in this manuscript:

ML	Machine learning
ResNet	Residual neural network
GANs	Generative adversarial networks
VAEs	Variational autoencoder
DRA	Dimensionality reduction algorithm
ISOMAP	Isometric mapping
t-SNE	t-distributed stochastic neighbor embedding
UMAP	Uniform manifold approximation and projection
PaCMAP	Pairwise controlled manifold approximation projection
DBSCAN	Density-based spatial clustering of applications with noise
ARI	Adjusted rand index
AI	Artificial intelligence

## Appendix A. Anomaly Samples

Anomaly Samples

## References

[1] Akhtar S., Tandiya A., Moussa M., Tarry C. (2019). An efficient automotive paint defect detection system. Adv. Sci. Technol. Eng. Syst. J..

[2] Kamani P., Afshar A., Towhidkhah F., Roghani E. Car body paint defect inspection using rotation invariant measure of the local variance and one-against-all support vector machine. Proceedings of the 2011 First International Conference on Informatics and Computational Intelligence.

[3] Kieselbach K.K., Nöthen M., Heuer H. (2019). Development of a visual inspection system and the corresponding algorithm for the detection and subsequent classification of paint defects on car bodies in the automotive industry. J. Coat. Technol. Res..

[4] Xu J., Zhang J., Zhang K., Liu T., Wang D., Wang X. (2020). An APF-ACO algorithm for automatic defect detection on vehicle paint. Multimed. Tools Appl..

[5] Armesto L., Tornero J., Herraez A., Asensio J. Inspection system based on artificial vision for paint defects detection on cars bodies. Proceedings of the 2011 IEEE International Conference on Robotics and Automation.

[6] Almhaithawi D., Bellini A. (2024). Defect Detection In Vehicle Painting: Case Study. Advances in Reliability, Safety and Security ESREL 2024 Collection of Extended Abstracts Part 1.

[7] Jiang Z., Hu X., Wang S. A mobile-transformer algorithm for car paint defect detection. Proceedings of the 2022 5th International Conference on Mechatronics, Robotics and Automation (ICMRA).

[8] Jiang Z., Hu X., Wang S. (2023). Image classification of car paint defect detection based on convolutional neural networks. Proceedings of the Journal of Physics: Conference Series, Volume 2456, The 2nd International Conference on Robotics, Automation and Intelligent Control (ICRAIC 2022), Changsha, China, 20–23 October 2022.

[9] Saberironaghi A., Ren J., El-Gindy M. (2023). Defect detection methods for industrial products using deep learning techniques: A review. Algorithms.

[10] Ciravegna G., Galante F., Giordano D., Cerquitelli T., Mellia M. (2024). Fault Prediction in Resistance Spot Welding: A Comparison of Machine Learning Approaches. Electronics.

[11] Bu H., Yang T., Hu C., Zhu X., Ge Z., Zhou H. (2024). An Image Classification Method of Unbalanced Ship Coating Defects Based on DCCVAE-ACWGAN-GP. Coatings.

[12] Geiß M., Baresch M., Chasparis G., Schweiger E., Teringl N., Zwick M. (2022). Fast and automatic object registration for human–robot collaboration in industrial manufacturing. Proceedings of the International Conference on Database and Expert Systems Applications.

[13] Cerquitelli T., Pagliari D.J., Calimera A., Bottaccioli L., Patti E., Acquaviva A., Poncino M. (2021). Manufacturing as a data-driven practice: Methodologies, technologies, and tools. Proc. IEEE.

[14] Velliangiri S., Alagumuthukrishnan S., joseph S.I.T. (2019). A review of dimensionality reduction techniques for efficient computation. Procedia Comput. Sci..

[15] Shen J., Wang R., Shen H.W. (2020). Visual exploration of latent space for traditional Chinese music. Vis. Inform..

[16] Crecchi F., Bacciu D., Biggio B. (2019). Detecting adversarial examples through nonlinear dimensionality reduction. arXiv.

[17] Tasoulis S., Pavlidis N.G., Roos T. (2020). Nonlinear dimensionality reduction for clustering. Pattern Recognit..

[18] Balasubramanian M., Schwartz E.L. (2002). The isomap algorithm and topological stability. Science.

[19] Van der Maaten L., Hinton G. (2008). Visualizing data using t-SNE. J. Mach. Learn. Res..

[20] McInnes L., Healy J., Melville J. (2018). Umap: Uniform manifold approximation and projection for dimension reduction. arXiv.

[21] Becht E., McInnes L., Healy J., Dutertre C.A., Kwok I.W., Ng L.G., Ginhoux F., Newell E.W. (2019). Dimensionality reduction for visualizing single-cell data using UMAP. Nat. Biotechnol..

[22] Wang Y., Huang H., Rudin C., Shaposhnik Y. (2021). Understanding how dimension reduction tools work: An empirical approach to deciphering t-SNE, UMAP, TriMAP, and PaCMAP for data visualization. J. Mach. Learn. Res..

[23] Ester M., Kriegel H.P., Sander J., Xu X. A density-based algorithm for discovering clusters in large spatial databases with noise. Proceedings of the KDD.

[24] Schubert E., Sander J., Ester M., Kriegel H.P., Xu X. (2017). DBSCAN revisited, revisited: Why and how you should (still) use DBSCAN. ACM Trans. Database Syst. (TODS).

[25] Liu F.T., Ting K.M., Zhou Z.H. Isolation forest. Proceedings of the 2008 Eighth IEEE International Conference on Data Mining.

[26] Chacón J.E., Rastrojo A.I. (2023). Minimum adjusted Rand index for two clusterings of a given size. Adv. Data Anal. Classif..

[27] Rousseeuw P.J. (1987). Silhouettes: A graphical aid to the interpretation and validation of cluster analysis. J. Comput. Appl. Math..

[28] Poli (2023). Final Year Car Paint Defect Dataset. https://universe.roboflow.com/poli-h7nww/final-year-car-paint-defect.

[29] He K., Zhang X., Ren S., Sun J. Deep residual learning for image recognition. Proceedings of the IEEE Conference on Computer Vision and Pattern Recognition.

[30] He K., Zhang X., Ren S., Sun J. (2016). Identity mappings in deep residual networks. Proceedings of the Computer Vision—ECCV 2016: 14th European Conference.

[31] Graves A., Graves A. (2012). Long short-term memory. Supervised Sequence Labelling with Recurrent Neural Networks.

[32] Vaswani A. (2017). Attention is all you need. Advances in Neural Information Processing Systems.

[33] Maintainers T., Contributors (2016). TorchVision: PyTorch’s Computer Vision library. https://github.com/pytorch/vision.

[34] Paszke A., Gross S., Massa F., Lerer A., Bradbury J., Chanan G., Killeen T., Lin Z., Gimelshein N., Antiga L. (2019). PyTorch: An Imperative Style, High-Performance Deep Learning Library. Advances in Neural Information Processing Systems 32.

[35] Pedregosa F., Varoquaux G., Gramfort A., Michel V., Thirion B., Grisel O., Blondel M., Prettenhofer P., Weiss R., Dubourg V. (2011). Scikit-learn: Machine Learning in Python. J. Mach. Learn. Res..

[36] McInnes L., Healy J., Saul N., Grossberger L. (2018). UMAP: Uniform Manifold Approximation and Projection. J. Open Source Softw..

